# At Our New Home

**Published:** 1881-06

**Authors:** 


### EDITORIAL DEPARTMENT.

"If thou hast truth to utter, speak;
And leave the rest to God."

At Our New Home.—Our regular readers were made aware, in the May issue of The Independent Practitioner, of its contemplated removal to the City of New York, and many of them understand and approve of the reasons and motives for transferring it from Baltimore to this city. To others we would say, that the advantages and facilities of the Metropolis of a great and rapidly-growing country like ours offer inducements to all legitimate enterprises which the smaller cities are unable to afford. Not that talent, energy and business capacity are lacking in less populous communities; but the fact that the aggregation of interests at a great centre like New York creates new channels for enterprise, and deepens and increases the capacity of existing ones far beyond what smaller populations are capable of doing. The Journal does not appear for the first time, and among strangers, in New York. Instead, some of its earliest and kindest friends are citizens of the new home in which it has elected to abide for the future. Its editors and business manager all have their friends in the Metropolis, and they cordially greet their confreres and cotemporaries, and promise to do all within their power to promote harmony and fraternal regard; and, while rendering even-handed justice to all, they hope to win an honorable place in the already distinguished list of New York journals devoted to medical science. Believing that there are verge and space enough in New York for one more addition to the anxious, earnest toilers in the old, yet ever new, science that looks to the mitigation of human suffering and the amelioration of the ills to which flesh is heir, The Independent Practitioner enters hopefully and cheerfully upon the performance of the duties it has voluntarily assumed, determined to *deserve* success in its new home.

